# Supplementary material for: Identifying the irrationality of the diagnosis of “pertussis-like syndrome” to enhance diagnostic accuracy
Source: Microbiol Spectr. 2025 Sep 23;13(11):e00737-25. doi: 10.1128/spectrum.00737-25 (PMC12584627; doi:10.1128/spectrum.00737-25)
Supplement: Table S1 — Distribution of the 10,561 cases in this study. [file spectrum.00737-25-s0001.doc]

**Table S1.** Distribution of the 10,561 cases in this study.

| Region | Province | Research centre | No. of cases |
| --- | --- | --- | --- |
| East China | Anhui | *Anhui Children’s Hospital | 544 |
|  | Fujian | Fuzhou Children’s Hospital of Fujian Province | 174 |
|  | Jiangsu | Children’s Hospital of Nanjing Medical University | 763 |
|  |  | *Children’s Hospital of Soochow University | 258 |
|  |  | Wuxi Children’s Hospital | 251 |
|  |  | Xuzhou Children’s Hospital | 132 |
|  | Jiangxi | *Jiangxi Provincial Children’s Hospital | 1253 |
|  |  | Jiujiang Maternity and Child Healthcare Hospital | 482 |
|  | Shandong | Children’s Hospital affiliated to Shandong University | 31 |
|  |  | Liaocheng Children’s Hospital | 13 |
|  | Zhejiang | Hangzhou Children’s Hospital | 30 |
| Northwest China | Gansu | *Gansu Provincial Maternity and Child-care Hospital | 294 |
|  | Ningxia | Yinchuan Women and Children Healthcare Hospital | 3 |
|  | Qinghai | *Qinghai Province Women and Children’s Hospital | 8 |
|  | Shaanxi | Xi’an Children’s Hospital | 175 |
|  | Xinjiang | Urumqi Children’s Hospital | 28 |
|  |  | *Xinjiang Children’s Hospital | 12 |
| North China | Beijing | Beijing Children’s Hospital, Capital Medical University | 17 |
|  | Hebei | Baoding Children’s Hospital | 246 |
|  |  | *Hebei Children’s Hospital | 831 |
|  | Inner Mongolia | *Inner Mongolia Maternity and Child Healthcare Hospital | 9 |
|  | Shanxi | *Children’s Hospital of Shanxi | 117 |
| Northeast China | Heilongjiang | Harbin Children’s Hospital | 497 |
|  | Jilin | Children’s Hospital of Changchun | 9 |
|  | Liaoning | Dalian Women and Children’s Medical Group | 6 |
|  |  | Liaoning Children’s Hospital | 2 |
| Central China | Henan | *Henan Children’s Hospital | 3320 |
|  | Hubei | Wuhan Children’s Hospital, Tongji Medical College, Huazhong University of Science & Technology | 50 |
|  | Hunan | *Hunan Children’s Hospital | 782 |
| Southwest China | Guizhou | Guiyang Children’s Hospital | 6 |
|  | Yunnan | Kunming Children’s Hospital | 200 |
| South China | Guangdong | Shenzhen Children’s Hospital | 17 |
|  | Guangxi | Liuzhou Maternity and Child Healthcare Hospital | 1 |

Note: *provincial hospitals
